# A Rare Case of Aggressive Systemic Mastocytosis With Skeletal Osteosclerotic Lesions on Presentation: A Diagnostic Conundrum

**DOI:** 10.7759/cureus.32135

**Published:** 2022-12-02

**Authors:** Rina Ghorpade, Fiona Tissavirasingham, Adarsh Vennepureddy

**Affiliations:** 1 Internal Medicine, Canton Medical Education Foundation, Aultman Hospital, Canton, USA; 2 Oncology, Aultman Hospital, Canton, USA

**Keywords:** computed tomography abdomen and pelvis, c-kit mutation, osteosclerotic lesions, mast cells, systemic mastocytosis

## Abstract

Systemic mastocytosis (SM) is a heterogeneous group of disorders caused by mast cell proliferation. SM often presents with non-specific symptoms making it a diagnostic challenge. Moreover, presentation with bone involvement is highly uncommon. Here, we report a rare case of SM in a 68-year-old female who initially presented with gastrointestinal symptoms and was later found to have sclerotic bone lesions on imaging. This case highlights an unusual presentation of SM, informing clinicians of the importance of keeping this disease process on the differential list of diagnostic conundrums.

## Introduction

Mastocytosis represents a rare hematological disease with malignant proliferation and excessive accumulation of neoplastic mast cells (MCs) in the bone marrow and various organ systems. Mastocytosis involving only the skin is known as cutaneous mastocytosis (CM), whereas the involvement of extracutaneous tissues is termed systemic mastocytosis (SM) [1]. The 2016 World Health Organization (WHO) classification of mastocytosis includes the following seven different types of mastocytosis: cutaneous mastocytosis, indolent systemic mastocytosis (ISM), smoldering systemic mastocytosis, systemic mastocytosis with an associated hematologic neoplasm (SM-AHN), aggressive systemic mastocytosis (ASM), mast cell leukemia, and mast cell sarcoma [2].

Clinically, SM can have a diverse presentation due to the chronic and intermittent release of MC mediators such as histamine, serotonin, tryptase, heparin, leukotrienes, prostaglandins, platelet-activating factor, proteases, and various cytokines, including tumor necrosis factor. Symptoms arising related to the release of MC mediators include urticaria, angioedema, anaphylactoid attacks, flushing and bronchospasm, digestive manifestations (nausea, vomiting, abdominal pain), and neuropsychiatric symptoms (depression, anxiety, memory disorders, sleeping disorders). Additionally, MC organ infiltration can result in dermatological manifestations such as urticaria pigmentosa; bone marrow disease including cytopenia; gastrointestinal (GI) involvement causing hepatosplenomegaly and malabsorption; and, rarely, skeletal system involvement leading to osteopenia, osteoporosis, and pathological fractures [3]. Moreover, skeletal lesions most commonly affect the axial skeleton, the proximal ends of long bones, and the pelvis. Common patterns of bone involvement include osteolysis and osteosclerosis. These lesions are often misdiagnosed as metastatic cancers, hyperparathyroidism, or Paget’s disease [4].

Due to the heterogeneous presentation of SM, diagnosis can be very challenging, especially in patients without cutaneous manifestations. Here, we present a case of aggressive SM who initially presented with GI symptoms and fatigue. For four to six months, the patient underwent extensive laboratory workup. After almost six months of diagnostic evaluation, she underwent multiple scans, including nuclear medicine bone scan, a magnetic resonance imaging (MRI brain), and various computed tomography (CT) scans. Finally, during around 9-12 months, she underwent various biopsies to arrive at the correct diagnosis of SM. This case report focuses on diagnostic difficulties related to SM and provides a brief review of the current knowledge of SM.

## Case presentation

A 68-year-old female with a medical history of hypothyroidism and chronic Darier’s disease under her left breast presented to the outpatient clinic with right upper quadrant abdominal pain for a three-week duration. The patient reported that the pain worsened after eating and was relieved with a heating pad. Additionally, she reported severe fatigue and decreased appetite. She denied nausea, vomiting, dysuria, constipation, diarrhea, or weight loss.

Lab workup showed normocytic anemia with a hemoglobin of 11.6 mg/dL, normal platelet count, normal white cell count, elevated erythrocyte sedimentation rate of 96 mm/hour (reference range (RR) = 0-29 mm/hour for women), and normal C-reactive protein of 2.4 mg/L (RR = 0-10mg/L). The comprehensive metabolic panel was only significant for a high alkaline phosphatase of 310 IU/L (RR = 44-147IU/L). The rheumatological workup was negative, including anti-nuclear antibodies, anti-nuclear cytoplasmic antibodies, mitochondrial, and anti-smooth muscle antibodies. Additionally, the hepatitis panel was also negative. The patient underwent serum and urine electrophoresis, which were negative for multiple myeloma. Tumor markers alpha-fetoprotein, cancer antigen 15-3, and cancer antigen 19-9 were negative. Tumor marker carcinoembryonic antigen was positive at 53.7 (RR = 0-38.6).

**Figure 1:**
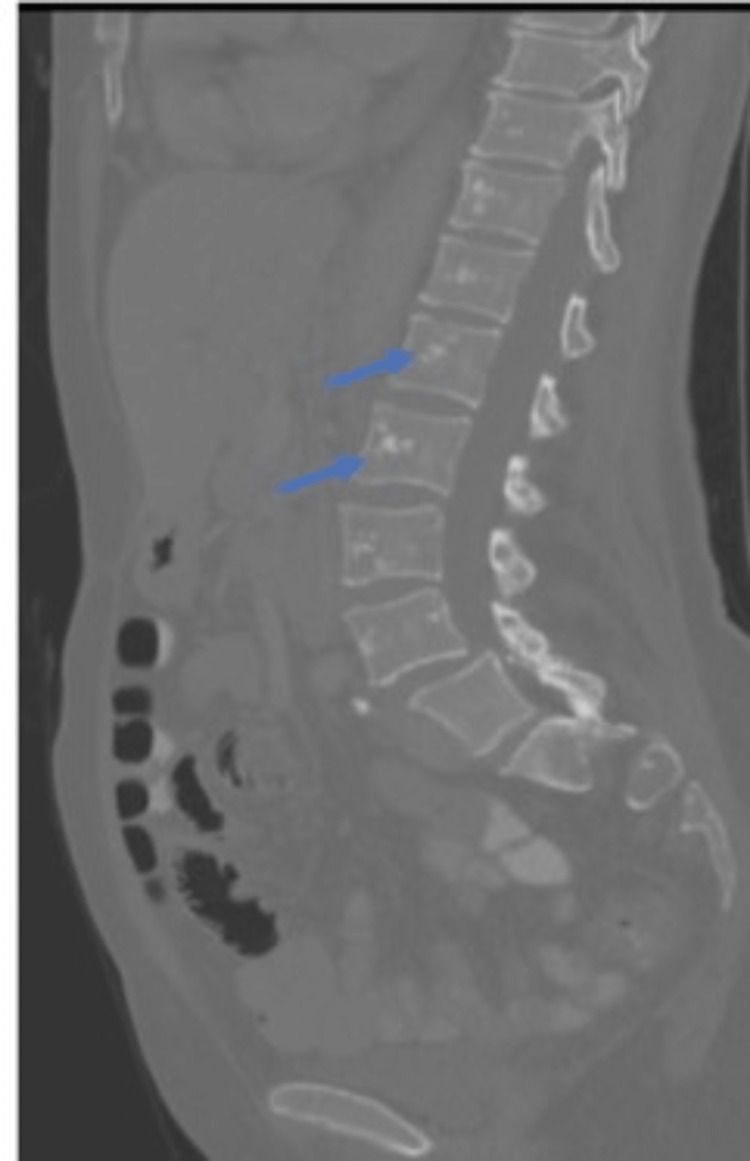
Computed tomography of the abdomen and pelvis showing multiple sclerotic lesions (blue arrows) in the thoracic and lumbar spine.

A right upper quadrant abdominal ultrasound (US) showed an over-distended gallbladder, with findings suggesting gallbladder sludge versus gallbladder polyp, along with common bile duct (CBD) dilatation. Moreover, the right upper quadrant US showed an echogenic liver lesion, most likely representing a hemangioma. Subsequently, the patient was referred to a gastroenterologist for a magnetic resonance cholangiopancreatography, which showed normal intrahepatic bile ducts, an 8 mm CBD at the porta hepatis (normal for the patient’s age), and no evidence of choledocholithiasis. A CT scan of the abdomen and pelvis was ordered to evaluate the patient’s persistent abdominal pain further, showing numerous scattered bony sclerotic lesions concerning for metastatic disease (Figure 1).

**Figure 2:**
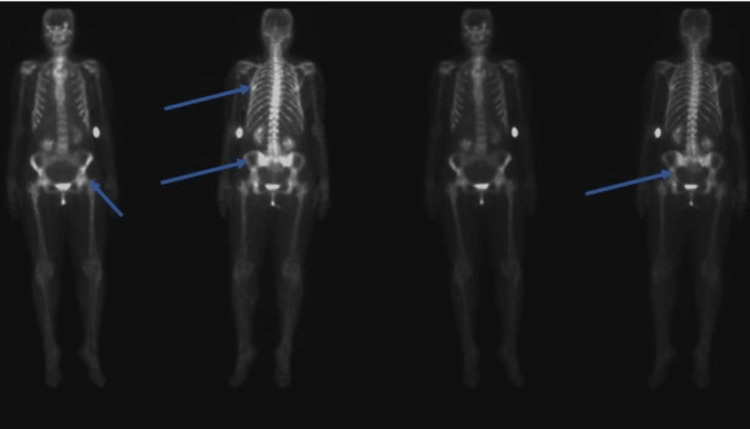
Nuclear medicine bone scan showing lytic lesions without focal uptake area of indeterminate etiology.

Additionally, there were calcific densities adjacent to the gallbladder wall measuring up to 1.2 cm, likely corresponding to a gallbladder polyp versus sludge. Numerous abdominal and retroperitoneal sub-centimeter lymph nodes were also noted. However, no pathologically enlarged lymph nodes were identified. Given the CT abdomen and pelvis findings, a nuclear medicine (NM) bone scan of the whole body was obtained, showing multiple areas of uptake involving the axial skeleton and proximal femurs, suspicious for metastatic disease (Figure 2).

In search of malignancies, a mammogram was ordered, and no mammographic evidence of malignancy was found. An MRI of the brain was obtained, which was negative for evidence of metastatic disease.

**Figure 3:**
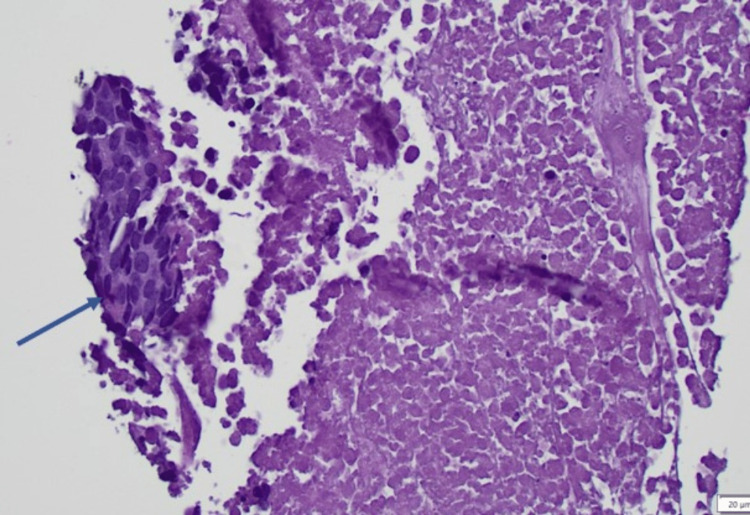
Left ischial bone biopsy showing small necrotic focus, with a minute cluster of atypical malignant cells at the 9 o’clock position (blue arrow) initially interpreted as metastatic carcinoma.

Over nine months into her evaluation, and due to the CT abdomen pelvis and NM bone scan findings suggestive of multiple bone metastasis, the patient was referred to oncology. Oncology recommended a bone biopsy of the left ischium. The pathology results of the bone biopsy showed that the morphological and histological features were consistent with metastatic carcinoma due to the presence of atypical cells (Figure 3).

**Figure 4:**
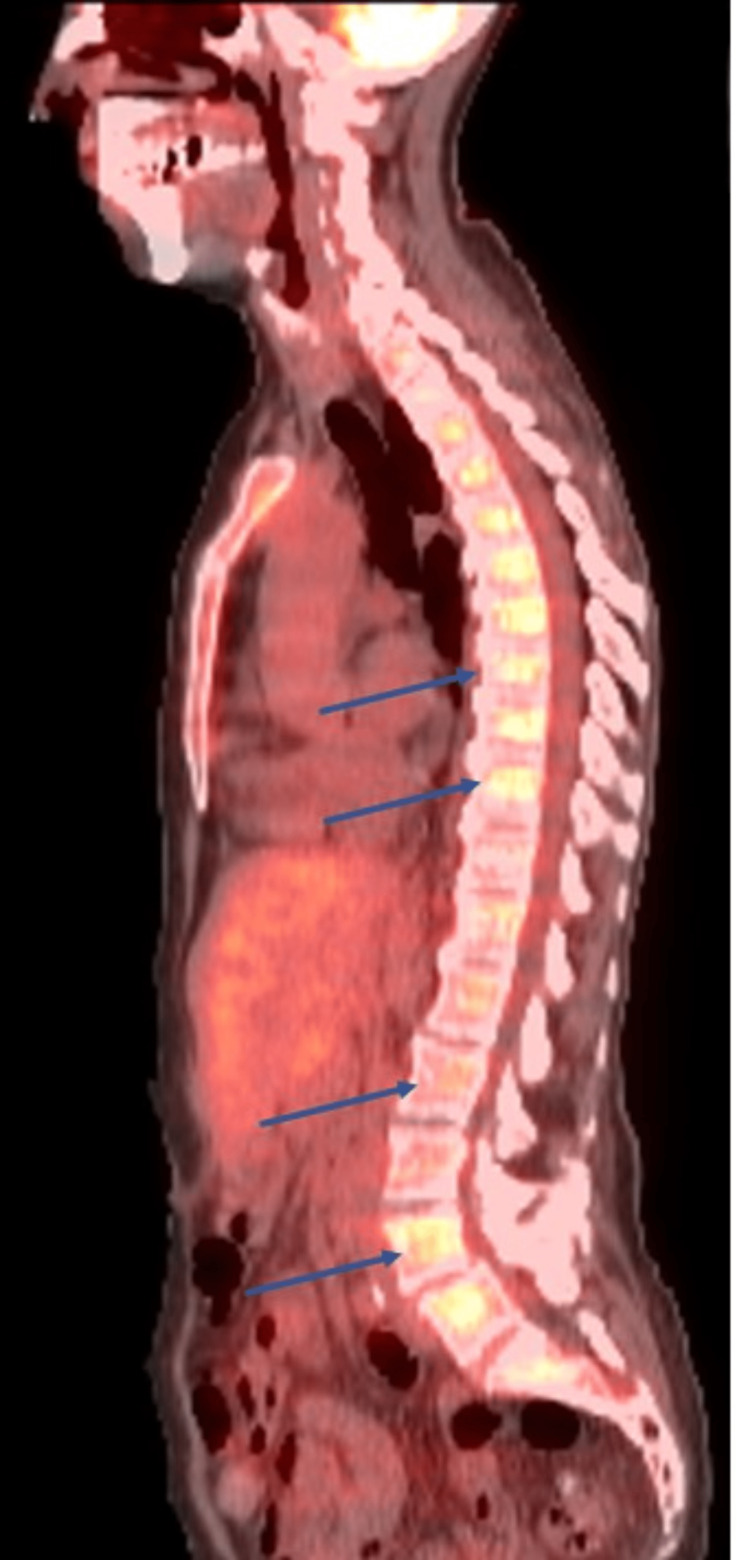
Fluorodeoxyglucose-positron emission tomography/computed tomography showing lytic lesions without focal uptake.

However, the immunohistochemical staining was negative, including for cytokeratin (CK) 7, CK20, thyroid transcription factor 1, GATA 3, p40, and pan keratin. Subsequently, fluorodeoxyglucose-positron emission tomography/CT skull to thigh was obtained, showing diffuse bone marrow uptake and a mild increase in splenic uptake, along with multiple lytic lesions without focal uptake (Figure 4).

**Figure 5:**
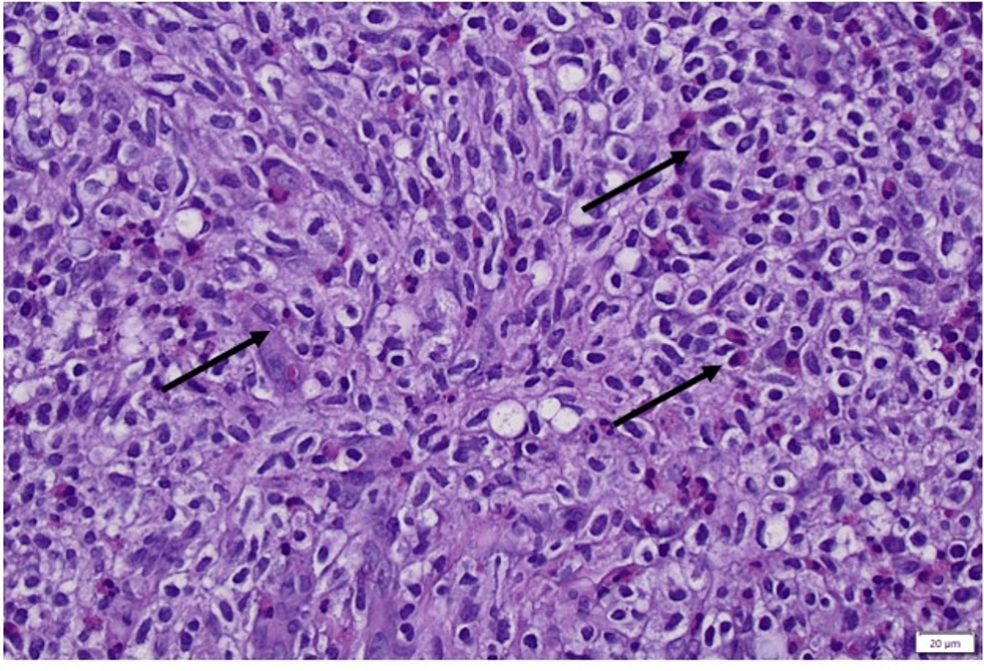
Bone marrow biopsy showing sheets of epithelioid and spindled mast cells (black arrows).

Due to the extensive workup findings, it was presumed that the patient had metastatic carcinoma of unknown primary. A bone marrow biopsy was performed by oncology which showed morphological findings consistent with SM representing 20% of hypercellular bone marrow and a subtle dyserythropoietic lineage of less than 10% suspicious for chronic myelomonocytic leukemia (CMML) (Figure 5).

**Figure 6:**
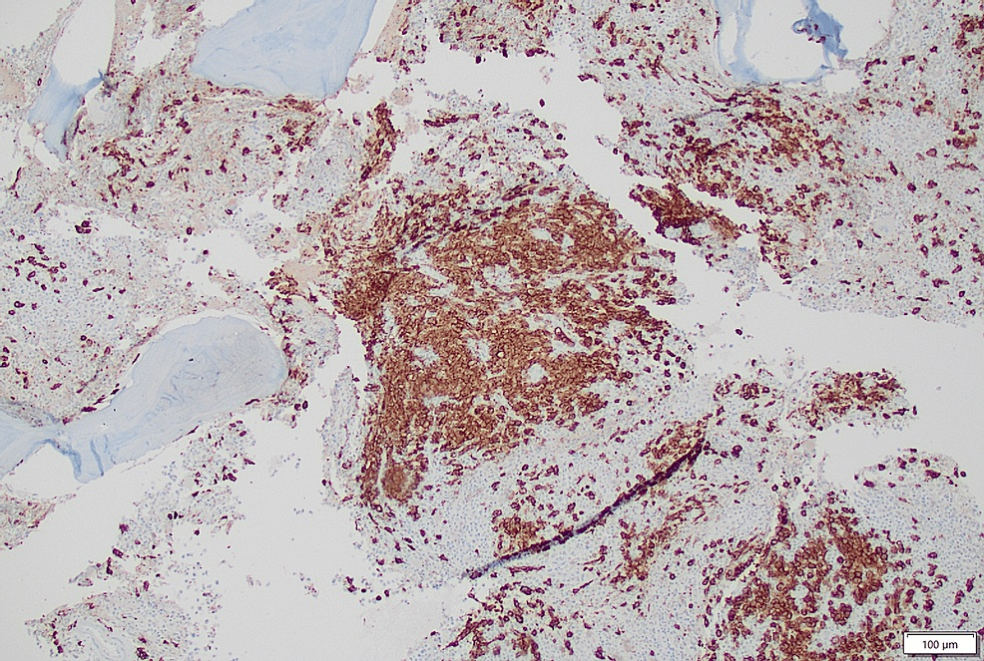
Bone marrow biopsy sample stained using immunostain for CD117 positivity in most mast cells.

However, this association was unclear, and correlation with cytogenetic/molecular studies to exclude BCR-ABL1 translocation was recommended. The *BCR-ABL1* mutation was negative, which ruled out the possibility of associated CMML. However, the *C-kit* mutation was positive for the *D816V* variant signifying SM. The bone marrow biopsy stain was strongly positive for CD117 stain (a transmembrane tyrosine kinase growth factor receptor that is a product of *c-kit* gene expression), suggesting the presence of MC disease (Figure 6).

**Figure 7:**
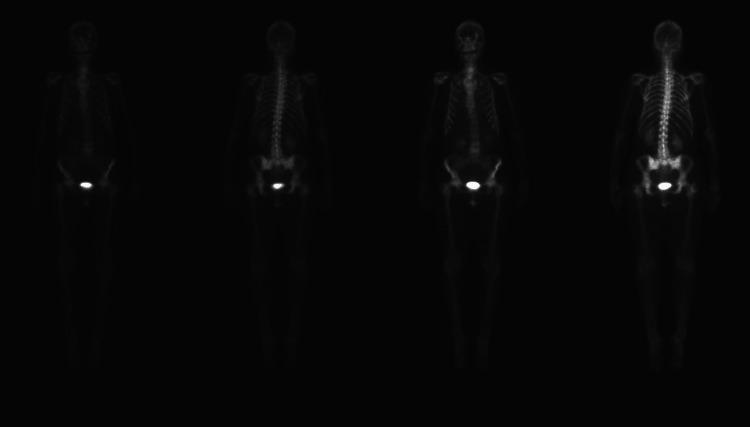
Nuclear medicine bone scan six months after starting midostaurin indicating reduced bone uptake.

After confirming the diagnosis of SM, tryptase level was obtained and found to be elevated at 174 ng/mL (RR = 1-30 ng/mL). The patient was started on midostaurin. A repeat bone scan after six months showed significant interval improvement (Figure 7).

After beginning treatment with midostaurin, her tryptase level also started to a downtrend, indicating decreased disease burden.

## Discussion

This case highlights the diagnostic dilemma that SM can present with, largely due to the ability of MCs to produce a highly variable systemic clinical presentation. MCs are derivatives of the myeloid lineage and are present in connective tissues throughout the body. Activation and degranulation of MCs are important processes that modulate various pathological and physiological processes such as vasodilation, angiogenesis, and innate and adaptative immunity responses. They are also important in allergic and anaphylactic reactions, wound healing, fibrosis, and autoimmune conditions [5]. They are derived from cluster of differentiation (CD) 34+, CD117+ (c-kit), and CD13+ progenitors. Proliferation, differentiation, and survival of MCs are under strict regulation by interleukins (IL)-3, IL-4, IL-9, and IL-10, stem cell factors (SCFs), and c-kit tyrosine growth factor receptor, which are expressed on the surface of MCs. Any abnormalities in c-kit receptors or regulation of SCFs lead to abnormal growth, differentiation, or apoptosis of MCs. Multiple *c-kit* mutations have been documented in cases of SM. However, the most common is the substitution of aspartate to valine in codon 816 (Asp816Val) or *D816* mutation, resulting in activation of the c-kit receptors and accumulation of MCs [6]. This mutation was present in this our patient.

SM is a heterogeneous and clonal disease with a myriad of manifestations due to the proliferation and accumulation of neoplastic MCs in various tissues and the release of various MC mediators. Bone involvement is present in about 5-11% of patients with SM and is usually associated with osteosclerotic lesions, osteoporosis, or both [7]. The diagnosis of SM is very challenging and is based on both histological and genetic factors, established using the WHO criteria. One major and one minor or three minor criteria are needed to establish the diagnosis [1]. Major criteria include multifocal dense infiltrates of MCs (>15 MCs in aggregates) in bone marrow biopsies and/or in sections of other extracutaneous organs. Minor criteria include >25% of MCs being atypical or spindle-shaped in MC infiltrates detected on sections of visceral organs; *c-kit* point mutation at codon 816 in bone marrow or another extracutaneous organ; MCs in bone marrow or blood or another extracutaneous organ exhibiting CD2 and/or CD25; and baseline serum tryptase level >20 ng/mL. Our patient met one major criterion of dense infiltrates of MCs in bone marrow biopsy, as well as two minor criteria of positive *c-kit* mutation in blood, and serum tryptase level of >20 ng/mL.

Treatment of SM is based on the individual subtype and is divided into two broad categories. The first is the anti-mediator therapy to inhibit bioactive mediators produced by MCs. The second is cytoreductive therapy which aims to reduce the disease burden. The anti-mediator therapy is sufficient for symptom management in ISM and SSM. It includes histamine (H1 and H2) receptor antagonists, MC stabilizers (cromolyn sodium), and leukotriene inhibitors [8]. In advanced variants (SM-AHN, ASM, and MCL), the anti-mediator therapy is concurrently used with cytoreductive therapy. Midostaurin is a multikinase inhibitor that inhibits the protein produced by c-kit, including the kinase encoded by mutated *D816V*. It received Food and Drug Administration (FDA) approval in 2017 for treating SM-AHN, ASM, and MCL, and it has been found to improve quality of life and mediator-related symptoms in patients with advanced SM [9]. The most common side effects of midostaurin are nausea and bone marrow suppression leading to pancytopenia. Our patient had the ASM subtype, and she responded well to midostaurin therapy.

Interestingly, in June 2021, the US FDA approved avapritinib for the treatment of SM. Avapritinib is another newer multikinase inhibitor with highly selective and potent activity against mutated c-kit and platelet-derived growth factor receptor A mutants, and was approved based on the results of two multicenter, open-label clinical trials (EXPLORER and PATHFINDER) for the treatment of advanced SM. The main side effects of avapritinib are edema, diarrhea, nausea, and fatigue. It is not recommended to treat patients with advanced SM with a platelet count of less than 50 × 10^9^/L due to the risk of intracranial hemorrhage [10].

## Conclusions

This case is unique in that the initial diagnostic workup had clinical and histopathological features similar to metastatic carcinoma with an unknown primary, misleading clinical subspecialties and pathologists alike. The patient underwent almost a full year of various diagnostic testing before finally confirming the correct diagnosis of SM. What made this case additionally challenging was that the patient did not have typical symptoms related to the release of MC mediators. Instead, she presented incidentally with abdominal symptoms leading to the initial CT scan of the abdomen and pelvis. This then prompted further evaluation, finally resulting in a bone biopsy, as well as a bone marrow biopsy, ultimately leading to the correct diagnosis of ASM.

SM can present with a myriad of non-specific symptoms which can be very debilitating to patients. This case highlights that due to a lack of obvious symptoms, arriving at the diagnosis of SM can be very challenging, causing diagnostic delays and increased patient frustration. Overall, this case represents a prime example of where keeping a broad differential for diagnostically challenging cases is crucial to arrive at an accurate diagnosis of SM and a tailored treatment plan.
